# Early Development of Newborn Screening for HCU and Current Challenges

**DOI:** 10.3390/ijns7040067

**Published:** 2021-10-25

**Authors:** Harvey L. Levy

**Affiliations:** 1 Division of Genetics and Genomics, Boston Children’s Hospital, 1 Autumn Str., Rm 526.1, Boston, MA 02115, USA; harvey.levy@childrens.harvard.edu; 2 Department of Pediatrics, Harvard Medical School, Boston, MA 02115, USA

**Keywords:** newborn screening, NBS, homocystinuria, HCU, methionine, homocysteine, false negative, missed cases, cystathionine b-synthase, CBS

## Abstract

Classic homocystinuria (HCU) was added to newborn screening (NBS) by Robert Guthrie a few years after the disorder was first described. The justification for NBS was similar to that for PKU, that presymptomatic identification and early dietary treatment would prevent the clinical consequences, which, for HCU, are mental deficiency, ectopia lentis, skeletal abnormalities, and thromboembolism. It was assumed that identifying increased methionine in the screening blood specimen would identify all affected neonates. However, it is now clear that many with HCU are missed by NBS, mainly because the methionine level in the first days of life is normal or below the cutoff level in the NBS program. This includes virtually all of those with B_6_-responsive HCU. Thus, a more effective method of NBS for HCU should be considered. Included among the possibilities are decreasing the methionine cutoff level, requiring an increase in the Met/Phe ratio if the methionine level is not at or greater than the cutoff level, using methionine as the primary screen with homocysteine as a second-tier test, or replacing methionine with homocysteine as the primary screen. Homocysteine is the primary metabolite that increases in HCU, while the methionine increase is secondary, so homocysteine is usually increased before the increase in methionine, almost always during the first few days of life. Finally, targeted gene screening might be considered. All of these possibilities would impose added expense and labor to NBS, so meeting these challenges would likely require a regional or national effort.

## 1. Introduction

The phone rang in the laboratory on a Friday morning in 1967; I picked up and the voice asked for Dr. Efron. I explained that Dr. Efron was quite ill and not expected in for some time. The voice explained that he was Dr. Robert MacCready, Director of the Massachusetts Newborn Screening Program, and that there was a newborn screening result that required medical evaluation. Could anyone from the lab come over to the screening program? I immediately said “yes”, and 10 min later was on my bike from the Massachusetts General Hospital in Boston heading for the State Laboratory Institute in the Jamaica Plain section of Boston, 7 miles away.

**Figure 1:**
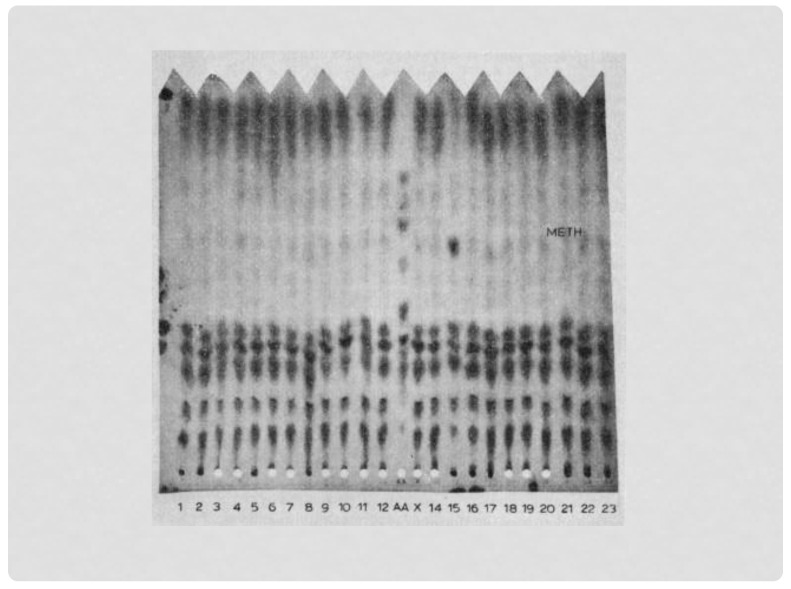
The increased methionine seen in the NBS specimen of the first baby with HCU identified in Massachusetts (#15). The screening method was one-way paper chromatography, the method used before adding the Guthrie bacterial assay for methionine in the Massachusetts program.

I was looking forward to seeing newborn screening (NBS). It was in a lecture by Dr. Mary Efron 2 years before that she explained the excitement surrounding newborn screening (NBS), how it could lead to the neonatal presymptomatic diagnosis of metabolic disease, and through early treatment prevent the intellectual disabilities and other features that ravaged these children and their families. Additionally, as a consultant to the Massachusetts Newborn Screening Program, she explained how valuable NBS was in identifying many more examples of these rare disorders than was possible from only clinical identification, and thus could lead to important research. It was after hearing this lecture that I decided to devote my genetics career to inborn errors of metabolism, and began my training with Dr. Efron at Massachusetts General Hospital. Tragically, it was due to Dr. Efron’s ultimately fatal illness during my second year of training that gave me the opportunity to learn firsthand what she had previously taught. I arrived at the State Laboratory Institute about an hour later and walked up to the third floor, where the Newborn Screening Program was located. After introductions, I was shown the abnormality, a big “spot” in a one-way chromatographic paper, indicating increased methionine in the blood of this baby (Figure 1). It was obvious that this baby had marked hypermethioninemia, and I had learned enough to know that the only metabolic disorder known at that time to be associated with increased methionine was homocystinuria (HCU). I immediately called the pediatrician indicated on the baby’s identification card, explained the finding, and asked if I could evaluate the baby at the hospital the following Monday. He agreed.

On Monday morning I saw the baby with his parents. He was clinically normal. I was aware that HCU was a genetic disorder with autosomal recessive inheritance, so I asked if they had other children and I was informed that they had a 4-year-old daughter who had some difficulty walking but was otherwise normal. After collecting blood and urine specimens from the baby to more thoroughly evaluate the reason for his increased methionine level, I asked the family to return with their daughter and baby in 2 days.

By the next day, it was evident that the baby had HCU. Amino acid analysis of plasma and urine revealed the presence of homocystine as well as increased methionine. The following day, I again saw the baby and this time his 4-year-old sister. It was immediately evident that the sister also had HCU. She was developmentally delayed, with only a few single words and no sentences. Her gait was wide-based and “duck waddle”, incorrectly ascribed to congenital hip dislocation, for which she was wearing several layers of diapers. Her face disclosed a malar flush and she seemed myopic, holding a book almost to her face in order to see the pictures.

Here I was, only a second-year fellow in research training, diagnosing the first case of HCU detected by NBS in the United States and one of the earliest cases of a child with clinical complications of HCU. Needless to say, this led to my lifelong involvement in and investigation of HCU, as well as NBS. No event in my professional life has been more significant.

## 2. Classic Homocystinuria (HCU)

At this time, HCU had only been recognized as a metabolic disorder for 5 years. It was discovered in 1962 by Carson and Neill during a survey of urine in mentally defective individuals in Northern Ireland [1], and specifically described by Carson and colleagues the following year [2]. Shortly afterwards, Carson and colleagues [3] and, in the United States, Schimke et al. [4] thoroughly described many cases, the latter by testing urine from those with ectopia lentis (dislocation of the ocular lenses) or assumed otherwise to have Marfan syndrome. Both of these publications pointed out the striking resemblance of HCU to Marfan syndrome, a well-known autosomal dominant genetic disease (Table 1). Meanwhile, Mudd and colleagues at the National Institute of Mental

**Figure 2:**
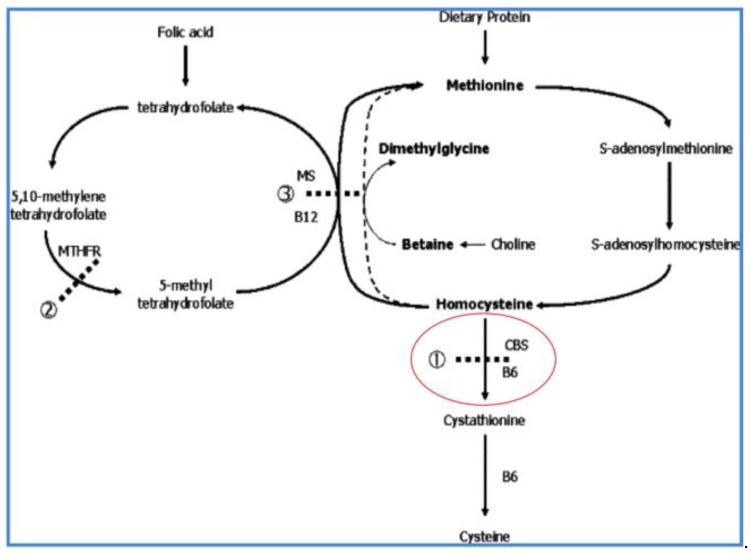
The methionine metabolic cycle showing the B_6_-dependent cystathionine β-synthase (CBS) deficiency in HCU. The transsulfuration conversion of homocysteine to cystathionine is at the junction of the cycle with transmethylation of methionine to homocysteine (on the right side of the cycle, as the figure is viewed), and remethylation to methionine on the left side of the cycle. CBS deficiency in HCU results in primary elevation of homocysteine and secondary elevation of methionine.

**Figure 3:**
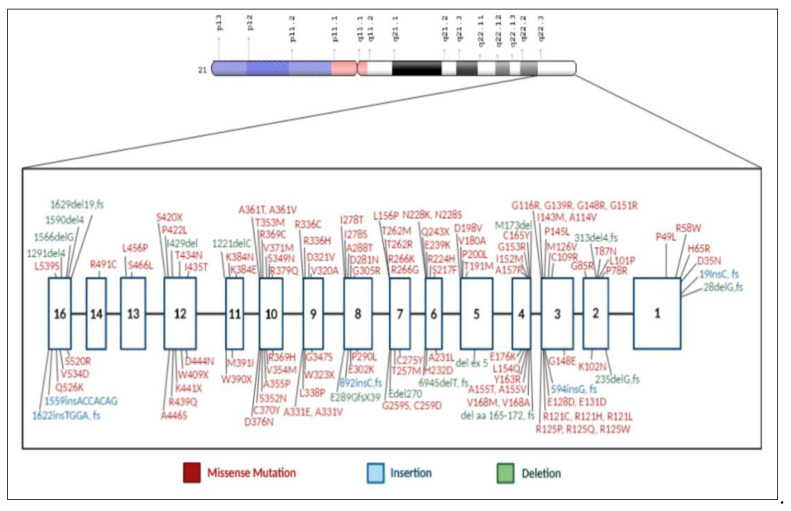
The gene for cystathionine β-synthase (CBS) and mutations associated with HCU. The gene has been mapped to chromosome 21q22.3. (Al-Sadeq, D.W.; Nasrallah, G.K., *Genes*, 2020, 11, 330; doi:10.3390/genes 11030330).

Health identified the metabolic block in HCU [5], the absence of detectable activity of cystathionine B-synthase (CBS), the transsulfuration enzyme in the methionine metabolic cycle that mediates the conversion of homocysteine to cystathionine (Figure 2). Thus, within 3 years of the discovery of HCU, its metabolic characteristics (increased homocysteine and increased methionine), its clinical abnormalities (intellectual disability, ectopia lentis, skeletal abnormalities, and thromboembolism), its resemblance to Marfan syndrome, and its enzymatic defect (CBS deficiency) all became known. Today, the full spectrum of clinical complications is recognized: mild intellectual reduction or frank disability, psychological or psychiatric manifestations, severe myopia that usually precedes lenticular dislocation (ectopia lentis), osteopenia or osteoporosis as well as Marfanoid habitus, and pulmonary embolus or stroke as manifestations of thromboembolism. However, clinical heterogeneity is most frequent among those with untreated HCU, and so the expressed abnormalities vary widely, some having only ectopia lentis with osteopenia but normal intelligence, others with the full Marfanoid habitus and stroke but normal lenticular placement, and others with still different combinations of abnormalities, and it is the recognition of one or more of the clinical features that leads to the diagnosis of HCU in those not identified by NBS. Following identification by NBS or suspicion on the basis of clinical expression, the diagnosis of HCU is made on the basis of its metabolic abnormalities and is usually confirmed by the presence of mutations in the CBS gene (Figure 3). Consult Sacharow et al. for a comprehensive description of HCU [6].

As noted in Figure 2, pyridoxal-5′-phosphate (vitamin B_6_) is the required cofactor for CBS. Consequently, there are two forms of HCU, B_6_-nonresponsive and B_6_-responsive. B_6_-nonresponsive HCU is associated with complete lack of detectable CBS activity in cultured fibroblasts and is usually accompanied by the development of the more severe clinical phenotype, while B_6_-responsive HCU is associated with detectable CBS activity and usually a milder phenotype.

### Treatment

**Figure 4:**
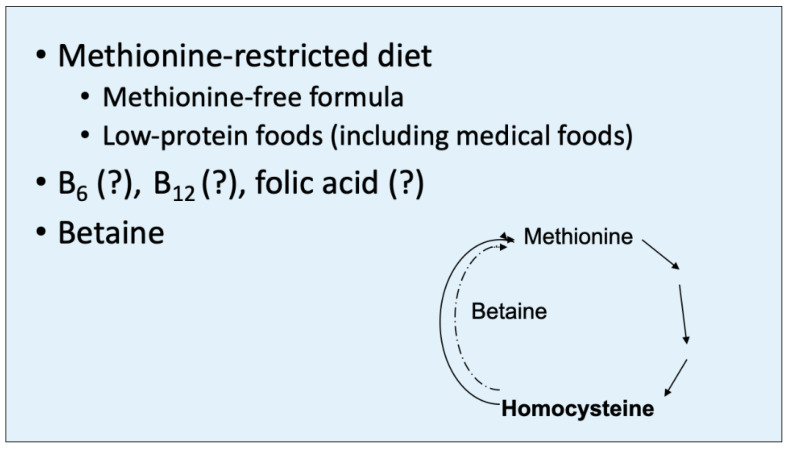
Betaine (trimethylglycine) contributes one of its methyl groups to homocysteine to aid in the remethylation of methionine, mediated by betaine-homocysteine transmethylase. This therapeutic mechanism serves to partially reduce the homocysteine level in HCU but increases the level of methionine. However, homocysteine appears to be the toxic metabolite in HCU, while increased methionine is largely benign.

Following the identification of HCU, a low-methionine diet is begun (Figure 4). The diet is markedly reduced in natural protein, so much so that protein restriction alone would quickly produce profound nutritional deficiencies. Consequently, a special medical food (often referred to as “formula”) is required. This “formula” is a methionine-free elemental mixture of essential and non-essential amino acids, carbohydrates, fat, essential fatty acids, minerals and vitamins to provide the additional required nutrition to support normal growth and development. For children beyond infancy and older individuals with HCU, a supplement known as betaine is also usually given. Betaine (trimethylglycine) is the substrate for betaine-homocysteine methyltransferase and stimulates the remethylation of methionine (i.e., it contributes one of its three methyl groups to homocysteine to form methionine, thus methylating homocysteine) (Figure 4). The goal of this diet and the betaine supplement is to reduce the markedly elevated concentration of homocysteine, presumably the toxic metabolite in HCU, and prevent clinical consequences.

For those found to be responsive to vitamin B_6_, a supplement of the vitamin, usually 100–300 mg, is also provided for treatment. An occasional individual with HCU is found to be so responsive to B_6_ that a supplement of this vitamin alone is sufficient to control the biochemical abnormalities, but this is very rare, and the vast majority of those with HCU, including those responsive to B_6_, require the diet and betaine supplement to one degree of another.

The objective of treatment is to reduce and maintain the level of total plasma homocysteine to <100 µmol/L (normal 10–15 µmol/L) from the untreated levels that are usually >100 µmol/L and often close to or above 200 µmol/L [7]. Studies in Ireland have demonstrated that for optimal benefit, the diet and control of homocysteine must begin in the neonate or very early in infancy, which almost always requires identification by NBS [8,9]. Unfortunately, NBS does not identify all affected babies, as will be discussed below, so treatment is often not initiated until clinical diagnosis. Nevertheless, treatment even after clinical problems are expressed may prevent further damage.

## 3. Newborn Screening for HCU

### 3.1. Early Development

By the late 1960s, the successful experience of PKU screening and early dietary treatment in preventing the clinical problems led to the idea that the same was true for other disorders, including HCU.

To accomplish this, Guthrie modified his bacterial inhibition assay so that it became responsive to additional metabolites, including an increased level of methionine as the marker for HCU [8]. This assay was added to NBS in a number of screening programs in the United States and in Ireland where HCU is relatively frequent, although it was many years before it became incorporated in all programs in the United States. Within a few years, it was evident that the promise of preventing clinical problems could be accomplished by NBS for HCU, just as developmental deficiency was prevented in PKU. A number of children identified by NBS and early treatment were growing and developing normally with normal vision, normal bone development, and without thromboembolitic events. In Ireland, 18 out of 21 persons between the ages of 2 and 23 years identified with HCU by NBS and compliant with dietary therapy had no complications [9]. The experience in Manchester, England was similar. Twelve persons with HCU identified by NBS and treated early had a median IQ of 100, compared to a median IQ of 58 for those diagnosed after infancy [10]. These results and many others have gradually led to the inclusion of HCU in the NBS menus. Inclusion in the United States was greatly enhanced by the designation of HCU as one of the 29 disorders in the uniform screening panel recommended by the American College of Medical Genetics [11]. Today, all NBS programs in the United States and many in other countries screen for HCU.

### 3.2. Missed Cases (False Negatives)

Despite the success of NBS for HCU, there has also been failure. Notably, babies with HCU have been missed by NBS. This is evident by those who present with the clinical characteristics of HCU, often misdiagnosed as having Marfan syndrome, and were considered normal in NBS. Many of these infants have the B_6_-responsive form of HCU, which accounts for up to 50% of HCU, is the more easily treated form of HCU, but is rarely identified by NBS [12,13]. But even those with B_6_-nonresponsive HCU, the most severely affected form, have been missed. For instance, at Boston Children’s Hospital, the three most recent cases of HCU we have encountered were misdiagnosed as “Marfan syndrome” for 17–23 years. All were B_6_-nonresponsive and had been screened for HCU as newborns with normal results. Many years ago, we encountered an infant with B_6_-nonresponsive HCU who presented at age 4 months with diet-induced folate deficiency. His NBS had also been considered normal [14]. Thus, it is clear that NBS can miss infants with HCU, including almost all with the B_6_-responsive form and probably many with B_6_-nonresponsive HCU. However, the number or percentage of missed cases of HCU is as yet unknown.

### 3.3. Reasons for Missing HCU in NBS

The most frequent reason for failure to identify HCU in NBS has been the omission in the NBS program. Although all NBS programs in the United States now include HCU in their menus, this has been true only since 2006, when the American College of Genetics recommended it in all NBS programs [11]. Some NBS programs in Europe do not include HCU screening, and only a few programs in other parts of the world include HCU [15].

The second most frequent reason that infants with HCU can be and have been missed is a normal methionine concentration in the newborn blood specimen or, likely more often, only a slight elevation of methionine below the cutoff level for detection in the program. The focus in NBS for HCU should now be on meeting this challenge.

## 4. Proposals to Reduce False Negatives in NBS

### 4.1. Decrease the Methionine Cutoff

When the Massachusetts program reduced the methionine cutoff from 134 to 67 µmol/L, the number of false negative results decreased (i.e., the sensitivity for detecting HCU was increased) [16]. However, this reduction in the methionine cutoff level also increased the false positive rate (i.e., reduced specificity), thereby resulting in the referral for evaluation of HCU of many more normal infants. Currently, a number of NBS programs have reduced the methionine cutoff level to as low as 45 µmol/L, just above the normal neonatal methionine level of approximately 34 µmol/L. To avoid the increase in false positive results (reduced specificity) that this reduced methionine cutoff would generate, these programs developed the following two methods for selecting babes for a clinical evaluation of HCU.

#### 4.1.1. Add the Methionine/Phenylalanine Ratio

The simplest way to reduce the number of false positive results (i.e., increase specificity) is to require a higher methionine level **or** an increased methionine/phenylalanine (Met/Phe) ratio or, as recommended by Huemer et al. [17], both.

#### 4.1.2. Second-Tier Total Homocysteine

A more complicated method is to add a second-tier determination of the total homocysteine concentration in the NBS specimen [18]. Thus, a specimen that met the criteria of increased methionine at or above the cutoff and/or an increased Met/Phe ratio would be tested by second-tier total homocysteine, and only those babies whose homocysteine is elevated would be referred for evaluation. This would not further increase sensitivity, since an elevated methionine level would likely be required but should substantially increase selectivity. However, measuring homocysteine in the NBS filter paper blood specimen requires the added expense of additional tandem mass spectrophotometric instrumentation (LC-MS/MS rather than only MS/MS) and additional labor. Only one NBS program in the United States, Mayo for Minnesota, performs this additional testing, and only a few programs send the specimens to Mayo as the referral laboratory for the second-tier test [19]. Nevertheless, second tier testing for other analytes in used in a number of NBS programs, and so is not out of the question for addition in NBS.

### 4.2. Primary Homocysteine Screening

Measuring total homocysteine as a primary screen would substantially increase sensitivity and specificity of NBS for HCU. In Qatar, with a uniquely high frequency of HCU (1:1800) due to the founder effect (virtually all affected babies harbor the R336C CBS variant), the primary screen for HCU is total homocysteine [20]. This would almost certainly greatly increase the sensitivity of NBS for HCU by identifying essentially all infants with B_6_-nonresponsive HCU and probably the majority of those with B_6_-responsive HCU, since it is expected that, with rare exceptions, all newborns with HCU will have increased homocysteine. Screening all newborn specimens as a primary (first-tier) screen as performed in Qatar, however, would substantially increase the cost and difficulty of NBS beyond the capability of virtually all other NBS laboratories. In the United States and Europe, this would require a number of regional referral laboratories or a national NBS laboratory.

### 4.3. Primary Genetic Screening for HCU

A potential unique approach would be adding primary screening of the most frequent CBS mutations to methionine. Of course, the most efficient use of this approach would be in a homogeneous population such as that in Qatar, where a founder effect has resulted in virtually a single mutation producing HCU, as noted above. In essentially all other populations, however, more than one mutation would have to sought so that a gene panel of mutant probes could be developed. In the European and American populations, the most frequent CBS mutation is the B_6_-responsive p.I278T. Close behind in frequency is the B_6_-nonresponsive “Celtic” variant p.G307S. In areas with relatively high populations of Spanish origin, the p.T191M variant could be added to the gene panel [21]. Several other mutations could also be added depending on the most frequent CBS genotypes known to be associated with HCU in the state or region. Therefore, the affected neonates whose methionine level is normal or near normal when the NBS blood specimen is collected could be identified. In addition, the most frequent genetic mutations associated with the remethylation disorders could also be added so that there would be coverage for all of the homocystinuric disorders.

However, there are at least two downsides of this approach. The first is the additional cost for genetic screening of every NBS specimen, both monetary and labor. This would likely be prohibitive for most NBS laboratories. The second is the likely large number of neonates who harbor only a single mutation represented in the panel. Virtually all of these neonates would be carriers, but an occasional such neonate might have HCU but not be identified as such because the second mutation would not be represented in the panel or would be a novel mutation. Moreover, there might be infants in whom both mutations are excluded from the panel or are novel. Thus, an occasional or rare false negative result might have to be tolerated in this proposal. Nevertheless, the larger number of affected infants identified by this proposal could more than compensate for the occasional missed infant. Therefore, this proposal might be seriously considered. These proposals and the relative sensitivities and specificities are listed in Table 2.

## 5. Conclusions

NBS for HCU using increased methionine levels misses a number of cases of HCU. These individuals are not diagnosed with HCU until after they develop symptoms, which are often irreversible, and in many instances after having been misdiagnosed as “Marfan syndrome”. Consequently, more effective methods for identifying neonates need to be considered. The challenge is to develop strategies for implementing these methods.

## Figures and Tables

**Table 1:** The major symptomatic features of HCU and Marfan syndrome. Note the similarity between the two genetic diseases, leading to the misdiagnosis of “Marfan syndrome” in many cases of HCU that were missed by NBS. However, there are key clinical differences between the two diseases, notably in the mental and cardiovascular manifestations.

	Homocystinuia	Marfan Syndrome
Ectopia Lentis	Yes	Yes
Tall Stature	Yes	Yes
Skeletal Abnormalities	Yes	Yes
Intellectual disability	Yes	No
Vascular occlusions	Yes	No
Mitral valve prolapse	No	Yes
Aortic root dilation	No	Yes

**Table 2:** Proposals to reduce false negatives in NBS for HCU, including the relative differences in sensitivities, sensitivities and difficulties.

	Sensitivity	Specificity	Difficulty
Decrease the MET cutoff	↑	↓↓	None
Decrease MET cutoff with MET/PHE ratio	↑↑	↑	Slight
Decrease MET cutoff with MET/PHE ratio + Second-tier homocysteine	↑↑	↑↑	High
Primary homocysteine	↑↑↑	↑↑	Very High
Primary genetic screening	↑↑↑	↑↓	High
